# Utilizing brown mushroom stem waste as an eco-friendly alternative to soybean meal in layer chick nutrition

**DOI:** 10.3389/fvets.2025.1637147

**Published:** 2025-08-11

**Authors:** Ahmed A. A. Abdel-Wareth, Kayla G. Stamps, Md Salahuddin, Venkatesh Balan, Maedeh Mohammadi, Woo Kyun Kim, Weihang Zhu, Ahmed A. Ahmed, Cassandra D. Gray, Adrian M. W. Aviña, Taylor Rounds-Carter, Trahmilla Carr, Amri Williams, Adebowale Bakare, Jayant Lohakare

**Affiliations:** ^1^Poultry Center, Cooperative Agricultural Research Center, Prairie View A&M University, Prairie View, TX, United States; ^2^Department of Animal and Poultry Production, Faculty of Agriculture, South Valley University, Qena, Egypt; ^3^Department of Engineering Technology, Biotechnology Program, Cullen College of Engineering, University of Houston, Sugarland, TX, United States; ^4^Department of Poultry Science, University of Georgia, Athens, GA, United States; ^5^Computer Science Department, Prairie View A&M University, Prairie View, TX, United States

**Keywords:** brown mushroom stem, sustainable poultry feed, soybean meal replacement, biochemical markers, gas emissions

## Abstract

**Objectives:**

This study evaluated the potential of brown mushroom stem (BMS) powder as a sustainable feed ingredient in poultry diets by assessing its effects on growth performance, health status, and environmental impact. Specifically, the research investigated whether the partial replacement of soybean meal with BMS powder could maintain productive performance while improving physiological responses and, gas emission in Lohmann LSL Lite chicks.

**Methods:**

The study involved 160 3-week-old Lohmann LSL Lite chicks. After a 4-day adaptation period on the control diet, the chicks were assigned to four dietary groups: 0% (control), 2%, 4%, and 6% BMS of replacing soybean meal. BMS was sourced from a local commercial mushroom producer. The stems were cleaned, washed, and freeze-dried to reduce their moisture content to below 5% by weight, ensuring extended shelf life. The freeze-dried BMS were then finely ground into powder for inclusion in the chicken feed. Each group consisted of five replicates, and each replicate had eight chicks. Feed intake (FI) and growth performance were recorded weekly over a 5-week duration in a cage system. Gas emissions from excreta were measured using a sensor-based system. At the end of the study, randomly selected birds were slaughtered for blood and organ collection for further analysis. Data analysis was performed using one-way ANOVA in SAS 9.4 software. Polynomial contrasts were used to analyze the linear and quadratic effects of increasing levels of BMS powder.

**Results:**

The results showed no significant (*P* < 0.05) differences in final body weight, weight gain, feed intake, and feed conversion ratio (FCR) among groups. Internal organ weight also showed no significant (*P* < 0.05) difference among groups, indicating the safety of BMS powder incorporation in chick diets. Blood biochemical parameters, including total protein, globulin, aspartate aminotransferase (AST), alkaline phosphatase (ALP), gamma-glutamyl transferase (GGT), uric acid, and cholesterol, exhibited both linear (*P* < 0.05) and quadratic (*P* < 0.05) effects with varying levels of BMS powder. Interestingly, gas emissions, including carbon dioxide (CO_2_), ammonia (NH_3_), and methane (CH_4_), measured on days 31 and 32 of the experiment, exhibited significant quadratic responses (*P* < 0.05) to increasing levels of BMS powder in the diet. While these effects were modest and limited to a short observation window, they suggest a potential short-term environmental benefit that warrants further investigation. These results indicate that BMS powder inclusion may positively influence certain biochemical markers and reduce the environmental footprint of poultry production.

**Conclusion:**

BMS powder could be a potential and sustainable replacement for soybean meal in poultry diets. It maintained consistent growth performance and organ weight, reduced gas emissions, and positively influenced blood biochemical markers, emphasizing its potential benefits. Future research should validate these findings in commercial settings and explore their long-term applications for broader adoption in eco-friendly production systems.

## 1 Introduction

As the global poultry industry confronts challenges related to antibiotic resistance, sustainability, and feed ingredient scarcity, there is an urgent need for innovative, eco-friendly feed alternatives that ensure animal health and productivity without relying on conventional additives. Among the promising candidates, edible mushrooms and their byproducts are increasingly recognized for their rich nutritional composition and valuable functional bioactive compounds (1, 2).

Mushroom species, particularly species such as *Agaricus bisporus* (Button) and *Pleurotus ostreatus* (Oyster), are natural source of polysaccharides, β-glucans, polyphenols, and terpenoid compounds known to promote antioxidant activity, modulate the immune system, and influence gut microbial dynamics (2). These bioactive compounds have been reported to support intestinal integrity, stimulate beneficial microbiota, and reduce inflammation, making them ideal candidates for natural growth promotion in livestock production (1, 3). Consequently, mushrooms have attracted interest both as functional foods for human consumption and as innovative feed ingredients in animal production systems.

Several studies have demonstrated that whole mushrooms or their extracts can positively influence poultry health status and production performance. For example, mushroom supplementation has been associated with improved feed efficiency, enhanced immune responses, reduced pathogen load, and better meat quality (4, 5). Moreover, studies on mushroom species like *Flamulina velutipes* (Enoki), and *Lentinula edodes* (Shitake) have shown potential effects on egg production and oxidative stability of poultry products, further reinforcing the value of mushrooms as valuable feed additives (6, 7). However, much of this research has focused on whole mushrooms or caps, while the stems (6%−10% of the whole mushroom) are often discarded during mushroom processing, remain underutilized. This oversight neglects their considerable nutritional potential. Button mushroom stems, particularly those from *Agaricus bisporus*, are rich in protein, fiber, vitamins, and minerals, and may contain comparable or even greater levels of bioactive compounds than the cap (8, 9). The valorization of such agricultural byproducts aligns with current trends in sustainable livestock production and circular agriculture, which aim to minimize waste and maximize resource efficiency. In this context, *spent mushroom substrate* (SMS), the residual biomass left after mushroom cultivation, has gained attention as a potential feed ingredient in animal nutrition. SMS consists of mushroom mycelium, unutilized substrate materials, and residual fruiting bodies, making it a source of fiber, polysaccharides, and bioactive compounds. Several studies have reported that the inclusion of SMS in poultry diets can improve growth performance, modulate gut microbiota, and enhance immune response, depending on its composition and inclusion rate (9, 10). These findings suggest that mushroom byproducts, including SMS and stem residues, can serve not only as alternative nutritional inputs but also as functional agents promoting poultry health and sustainability.

This study investigates the potential of using freeze-dried brown mushroom stems (BMS) powder derived from *Agaricus bisporus* as a partial replacement for soybean meal in the diets of Lohmann LSL Lite layer chicks. Soybean meal, though a common protein source in poultry diets, is linked to several sustainability concerns, including deforestation, high water consumption, and economic instability due to fluctuating global prices. In contrast, BMS is a nutrient-rich, low-cost, and underutilized byproduct of the mushroom industry that is readily available and often discarded as waste. Its incorporation into poultry feed represents a promising strategy to reduce environmental impact while enhancing resource efficiency. By exploring the use of BMS as an alternative protein source, this study aims to support sustainable poultry production systems. The research evaluates key indicators such as growth performance, feed intake (FI) and conversion efficiency, internal organ development, gas emissions from excreta, and blood biochemical parameters to determine the nutritional and functional viability of BMS in poultry nutrition.

## 2 Materials and methods

The study was conducted at the Poultry Center of Prairie View A&M University (PVAMU), Prairie View, TX, USA. All animal handling and experimental procedures adhered to the Animal Welfare Act (AWA) and Animal Welfare Regulations (AWRs) established by the United States Department of Agriculture (USDA), as well as the Guide for the Care and Use of Laboratory Animals. Ethical approval was obtained from the Institutional Animal Care and Use Committee (IACUC) of PVAMU under protocol number AUP# 2023-055.

### 2.1 Animals, experimental design, and feed management

A total of 160 1-day-old Lohmann LSL Lite layer chicks were obtained from Hy-Line North America, LLC (Bryan, TX, USA). For the first 16 days, birds were fed a standard commercial starter diet and then transitioned to the control diet for a 4-day adaptation period prior to the beginning of the experimental feeding. The 3-week-old chicks were randomly assigned to four dietary treatments: a control group (0% BMS) and three treatment groups where 2%, 4%, or 6% of soybean meal was substituted with BMS powder. Each treatment consisted of five replicates with eight chicks per replicate. The experiment lasted 36 days.

The BMS powder was prepared using only the freeze-dried BMS, which were collected from a local mushroom farm (Monterey Mushrooms, Madisonville, TX, USA). The stems were washed, freeze-dried using a Harvest Right freeze dryer (North Salt Lake, UT, USA), and ground into fine powder. Experimental diets were formulated to be isonitrogenous and isocaloric, meeting or exceeding the nutrient requirements for layer chicks (11). Diets were prepared at the Texas A&M University Feed Mill (College Station, TX, USA) and provided in crumble form (Table 1). The chemical composition, amino acid profile, and nutritional value of BMS are presented in Table 2. Feed and water were offered *ad libitum* throughout the study. Weekly feed supply was adjusted by subtracting residual feed to monitor feed intake accurately. The birds were housed in an environmentally controlled brooder room in wire battery cages (GQF Manufacturing, Savannah, GA, USA) with five tiers and individual waste collection trays. A 24-h light cycle and recommended temperature ranges were maintained throughout the experiment.

**Table 1:** Ingredient and chemical composition of the experimental diets using brown mushroom stem powder fed to chicks.

		**BMS powder**		
**Ingredients %**	**Control**	**2%**	**4%**	**6%**
Corn	66.000	66.000	66.000	66.000
Soybean meal 48%	25.887	25.369	24.852	24.334
Brown mushroom stems 19.63% CP	0.000	0.518	1.035	1.553
Soybean oil	1.000	1.000	1.000	1.000
Vitamin^a^	0.050	0.050	0.050	0.050
Trace mineral^a^	0.100	0.100	0.100	0.100
Limestone	2.200	2.200	2.200	2.200
Mon-calcium phosphate	1.733	1.733	1.733	1.733
Salt	2.700	2.700	2.700	2.700
Sodium bicarbonate	0.133	0.133	0.133	0.133
DL-methionine	0.197	0.197	0.197	0.197
Total (kg)	100	100	100	100
**Calculated chemical compositions**				
ME (MJ/kg)	11.798	11.782	11.767	11.751
Crude protein (%)	18.036	17.889	17.742	17.595
Crude fiber %	2.358	2.389	2.414	2.442
Crude fat %	3.767	3.767	3.768	3.768
Calcium (%)	1.199	1.098	1.097	1.095
Phosphorus (%)	0.746	0.743	0.742	0.737
Lysine (%)	0.953	0.941	0.929	0.917
Methionine (%)	0.466	0.463	0.460	0.456

a The vitamin–mineral premix was supplemented at the following levels per kilogram of diet: 8,818,342 IU of vitamin A; 3,086,420 IU of vitamin D3; 36,742 IU of vitamin E; 13 mg of vitamin B1_2_; 1,177 mg of menadione; 4,775 mg of riboflavin; 16,168 mg of D-pantothenic acid; 2,350 mg of thiamine; 36,742 mg of niacin; 5,732 mg of vitamin B6; 1,398 mg of folic acid; 104,460 mg of choline; 441 mg of biotin; 120 mg of manganese (Mn); 1.4 mg of copper (Cu); 120 mg of zinc (Zn); 120 mg of iron (Fe); 0.5 mg of selenium (Se); and 800 mg of iodine (I).

WBMSP, Washed Brown Mushroom stem powder.

**Table 2:** Proximate and amino acid analysis of brown button mushroom stems.

Analysis^*^	**Brown mushroom stem**
Moisture %	10.50 ± 0.17
Dry matter %	89.50 ± 0.17
Crude protein %	19.63 ± 0.06
Crude fiber %	8.92 ± 0.12
Crude fat %	1.07 ± 0.06
Ash %	5.80 ± 0.00
Aspartic acid %	1.11 ± 0.28
Threonine %	0.57 ± 0.14
Serine %	0.525 ± 0.07
Glutamic acid %	1.895 ± 0.07
Proline %	0.54 ± 0.00
Glycine %	0.565 ± 0.07
Alanine %	0.73 ± 0.14
Cystine %	0.165 ± 0.35
Valine %	0.615 ± 0.21
Methionine %	0.19 ± 0.00
Isoleucine %	0.53 ± 0.00
Leucine %	0.805 ± 0.07
Tyrosine %	0.44 ± 0.00
Phenylalanine %	0.545 ± 0.07
Lysine %	0.685 ± 0.07
Histidine %	0.27 ± 0.14
Arginine %	0.595 ± 0.21
Tryptophan %	0.255 ± 0.78

* The tests were repeated three times. The values are expressed as the mean ± standard deviation.

### 2.2 Growth performance evaluation

Growth performance parameters were assessed throughout the entire experimental period, which lasted for 35 days. All chicks were individually weighed on day 1 (initial body weight) and again on day 35 (final body weight) using a precision digital scale (±0.01 g accuracy). These measurements were used to calculate average body weight gain per bird within each replicate cage. Feed intake (FI) was recorded on a weekly basis. The amount of feed offered to each replicate was recorded at the beginning of each week, and residual feed was collected and weighed at the end of the week. Weekly feed intake was calculated by subtracting the remaining feed from the amount initially provided. The cumulative feed intake over the 35-day trial was calculated per bird's average feed intake. Feed conversion ratio (FCR), an indicator of feed efficiency, was calculated for each replicate by dividing the total feed intake (g) by the total body weight gain (g) over the entire experimental period.

### 2.3 Sample collection and organ weight measurement

After completing the feeding trial, two birds from each replicate (*n* = 10 per treatment) were randomly selected and fasted for 12 h prior to humane slaughter on day 36. After slaughter, birds were manually eviscerated, and the liver, heart, spleen, and gizzard were removed and weighed. Relative organ weights were calculated as a percentage of live body weight.

### 2.4 Blood collection and serum biochemistry

At the end of the 35-day feeding trial, two birds per replicate (*n* = 10 birds per treatment group) were randomly selected for blood sampling to evaluate various serum biochemical parameters. The birds were not subjected to feed withdrawal prior to sample collection to avoid stress-related alterations in blood metabolites. Approximately 5 ml of blood was collected from the jugular vein using sterile 22-gauge needles and disposable syringes. Following collection, blood samples were immediately transferred into sterile serum separator tubes (BD Vacutainer^®^, Franklin Lakes, NJ, USA) and kept undisturbed to clot at room temperature (~22°C) for 45–60 min. After clotting, the tubes were centrifuged at 2,000 × g for 15 min using Sorvall ST Plus centrifuge (Thermo Electron LED GmbH, Langenselbold, Germany) to separate the serum. The resulting clear serum was carefully pipetted into 1.5 ml sterile microcentrifuge tubes, aliquoted to avoid multiple freeze-thaw cycles, and stored at −20°C until biochemical analyses were performed. All serum samples were subsequently transported on dry ice to the Arkansas State Veterinary Diagnostic Laboratory (Little Rock, AR, USA) for biochemical analysis. The panel of tests included the evaluation of major blood metabolites and clinical chemistry indicators: total protein, albumin, globulin, aspartate aminotransferase (AST), alkaline phosphatase (ALP), gamma-glutamyl transferase (GGT), total bilirubin, amylase, cholesterol, uric acid, phosphorus, calcium, sodium, potassium, and chloride. These parameters were selected to provide a comprehensive assessment of protein metabolism, liver and kidney function, enzymatic activity, and electrolyte balance in response to dietary inclusion of BMS powder. All procedures were carried out under strict aseptic conditions to ensure the samples' integrity and the biochemical evaluations' accuracy. The results were used to assess layer chicks' physiological and metabolic responses to increasing levels of BMS powder in the diet.

### 2.5 Gas emissions

**Figure 1:**
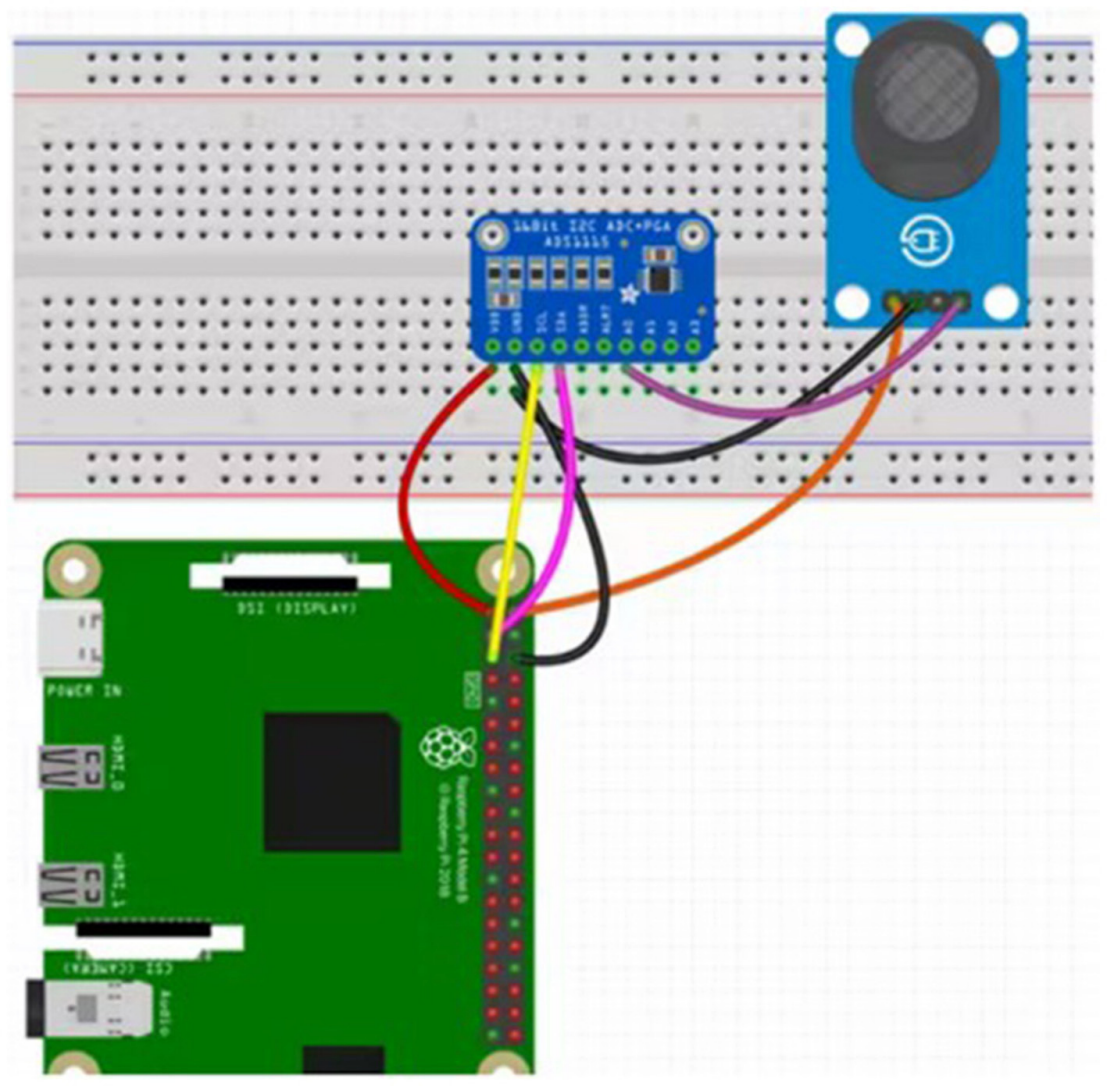
A schematic figure showing the design of the IoT-based system. Schematic setup of the gas sensor system using a Raspberry Pi 5, ADS1115 analog-to-digital converter module, MQ-135 gas sensor, jumper wires, and a breadboard.

On days 31 and 32, gaseous emissions from the excreta including carbon dioxide (CO_2_), ammonia (NH_3_), and methane (CH_4_), were measured using an Internet of Things (IoT)-based sensor system. The system comprised a Raspberry Pi microcontroller, an ADS1115 analog-to-digital converter module, an MQ-135 gas sensor, jumper wires, and a breadboard, as illustrated in Figure 1. The MQ-135 sensor was calibrated and configured to detect CO_2_, NH_3_, and CH_4_ gas emissions. Gas emission measurements were conducted under controlled conditions, using the same cages, environment, and time of day across all replicates to ensure consistency. Emissions were recorded at the tray level for each replicate cage, with data collection lasting 2 min per cage.. Real-time gas sensor readings were captured and stored digitally using the Raspberry Pi system. The data were transmitted to and archived in a central database maintained by computer scientists at the Department of Computer Science, Prairie View A&M University for analysis.

### 2.6 Chemical composition

The chemical composition of the experimental diets and brown mushroom stem samples was determined at the laboratories of Houston University following standard procedures described by the Association of Official Analytical Chemists (12). Dry matter (DM) was determined by oven-drying at 105°C according to AOAC Method 930.15. Ash content was measured via incineration in a muffle furnace at 550°C following AOAC Method 942.05. Crude protein (CP) was analyzed using the Kjeldahl method (AOAC Method 984.13), and ether extract (EE) was determined using the Soxhlet extraction technique (AOAC Method 920.39). Additionally, crude fiber was assessed using the Weende method as described by AOAC. Amino acid composition was determined according to the AOAC 994.12 method with the help of Midwest laboratories, Inc. Gretna, NE, USA.

### 2.7 Statistical analysis

Data were analyzed using the General Linear Model (GLM) procedure in SAS version 9.2 (13) (SAS Institute Inc., Cary, NC, USA) based on a completely randomized design. Treatment was considered a fixed effect. To evaluate the effects of increasing dietary BMS powder levels, orthogonal polynomial contrasts were used to assess linear and quadratic trends. Means were compared using Duncan's Multiple Range Test, with statistical significance set at *P* < 0.05. Results are reported as means ± standard error of the mean (SEM). For growth performance and gas emission measurements, five replicate pens per treatment were used, with each pen serving as an experimental unit. This replication level ensured adequate statistical power and is consistent with previous nutritional studies in poultry, allowing detection of biologically meaningful differences among dietary treatments. For organ weights and blood biochemical analyses, 10 birds per treatment group were sampled. This sample size balances statistical rigor with ethical considerations, and it is sufficient to detect treatment-related physiological changes, as supported by established protocols in poultry research. All figures were created using GraphPad Prism version 9 (GraphPad Software, La Jolla, CA, USA).

## 3 Results

### 3.1 Growth performance

Growth performance data are presented in Table 3. No statistically significant differences (*P* < 0.05) were observed among the treatment groups in terms of final body weight, body weight gain, feed intake, or feed conversion ratio. However, a trend toward reduced body weight gain was noted with increasing levels of BMS supplementation, with the *P*-value approaching significance (*P* = 0.053).

**Table 3:** Effects of the brown mushroom stem powder on growth performance of chicks.

**Inclusion level (%)**	**Initial BW, g/bird^−1^**	**Final BW, g/bird^−1^**	**BW gain, g/bird^−1^**	**Feed intake, g/bird^−1^**	**FCR**
Control	176.22	683.37	507.15	680.47	1.34
2% Mushroom stem	176.42	680.70	504.27	649.25	1.29
4%Mushroom stem	176.42	672.28	495.86	652.01	1.31
6% Mushroom stem	176.57	669.87	493.30	641.31	1.30
SEM	1.362	5.612	5.360	17.457	0.034
***P*** **-value**					
Linear^a^	0.865	0.069	0.053	0.161	0.527
Quadratic^b^	0.986	0.981	0.977	0.565	0.598

a Linear responses to dietary inclusion mushroom stem levels.

b Quadratic responses to dietary inclusion mushroom stem levels.

BW, body weight at 3 weeks of age; Final BW, body weight at 8 weeks of age; Feed intake, total feed intake during the experimental period, 5 weeks; FCR, feed conversion ratio; SEM, standard error of the means.

### 3.2 Internal organs

Organ weight data are presented in Table 4. There were no significant differences (*P* > 0.05) in the relative weight of most internal organs, including the liver, spleen, and gizzard among the dietary groups. However, heart weight exhibited a significant linear increase (*P* < 0.05) with increasing levels of BMS inclusion in the diet.

**Table 4:** Effects of the brown mushroom stem powder on relative internal organs weight of chicks.

**Inclusion level (%)**	**LBW, g**	**Heart %**	**Liver %**	**Spleen %**	**Gizzard %**
Control	694.72	0.62	2.15	0.28	3.03
2% Mushroom stem	681.92	0.55	2.16	0.30	3.10
4% Mushroom stem	696.04	0.63	2.11	0.26	2.88
6% Mushroom stem	696.86	0.68	2.12	0.32	2.91
SEM	13.926	0.025	0.063	0.014	0.098
***P*** **-value**					
Linear^a^	0.743	0.023	0.687	0.298	0.201
Quadratic^b^	0.628	0.021	0.993	0.131	0.845

a Linear responses to dietary inclusion of mushroom stem levels.

b Quadratic responses to dietary inclusion of mushroom stem levels.

LBW, live body weight; SEM, standard error of the means.

### 3.3 Blood parameters

**Figure 2:**
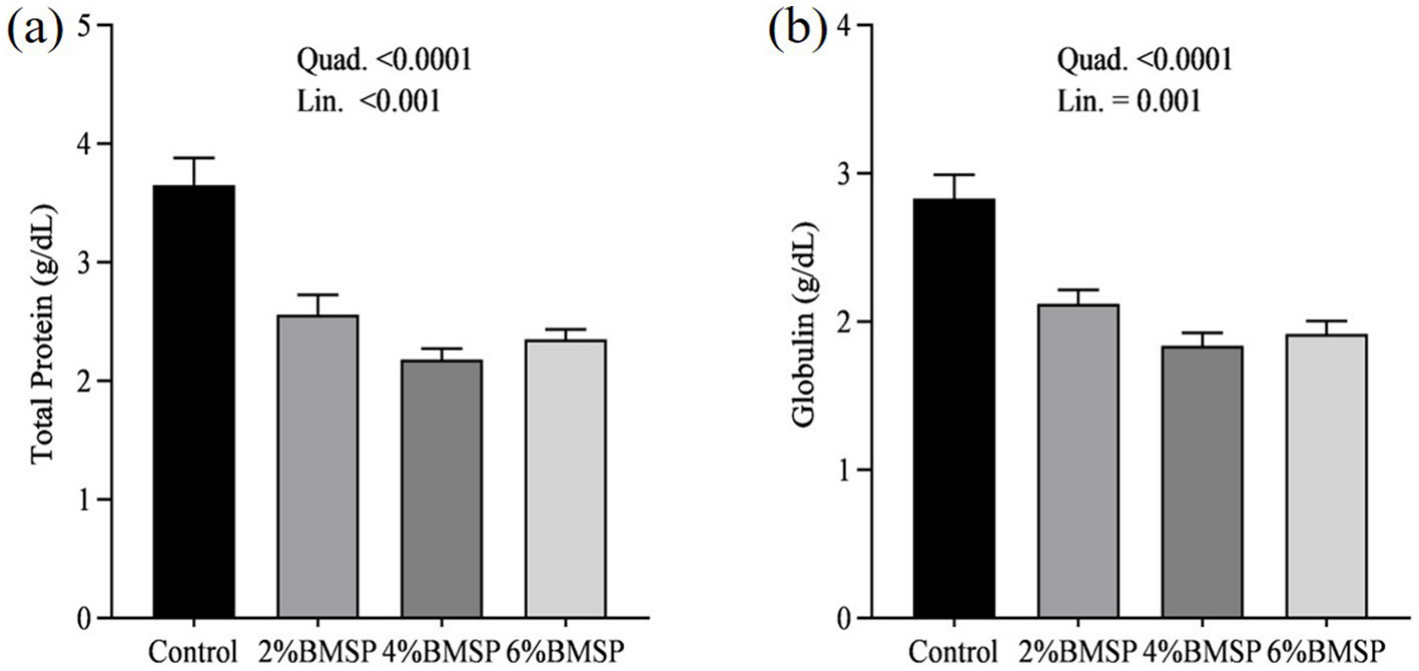
Effects of dietary brown mushroom stem powder (BMSP) supplementation on serum total protein **(a)** and globulin **(b)** concentrations in layer chicks. Lin, linear response; Quad, quadratic response; 2% BMSP, 4% BMSP, 6% BMSP, diets containing 2%, 4%, or 6% BMSP, respectively.

**Figure 3:**
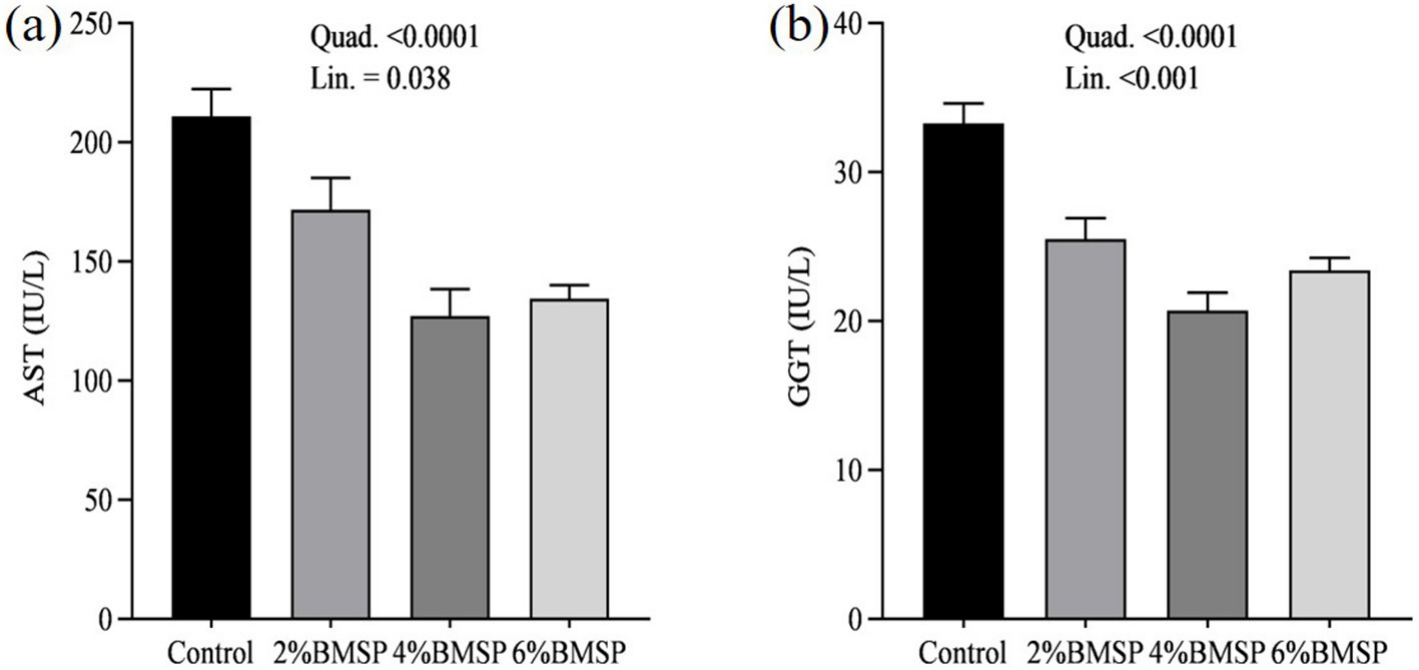
Effects of dietary brown mushroom stem powder (BMSP) supplementation on liver enzyme activities, including aspartate aminotransferase (AST, **a**) and gamma-glutamyl transferase (GGT, **b**), in layer chicks. Lin, linear response; Quad, quadratic response; 2% BMSP, 4% BMSP, 6% BMSP, diets containing 2%, 4%, or 6% BMSP, respectively.

**Figure 4:**
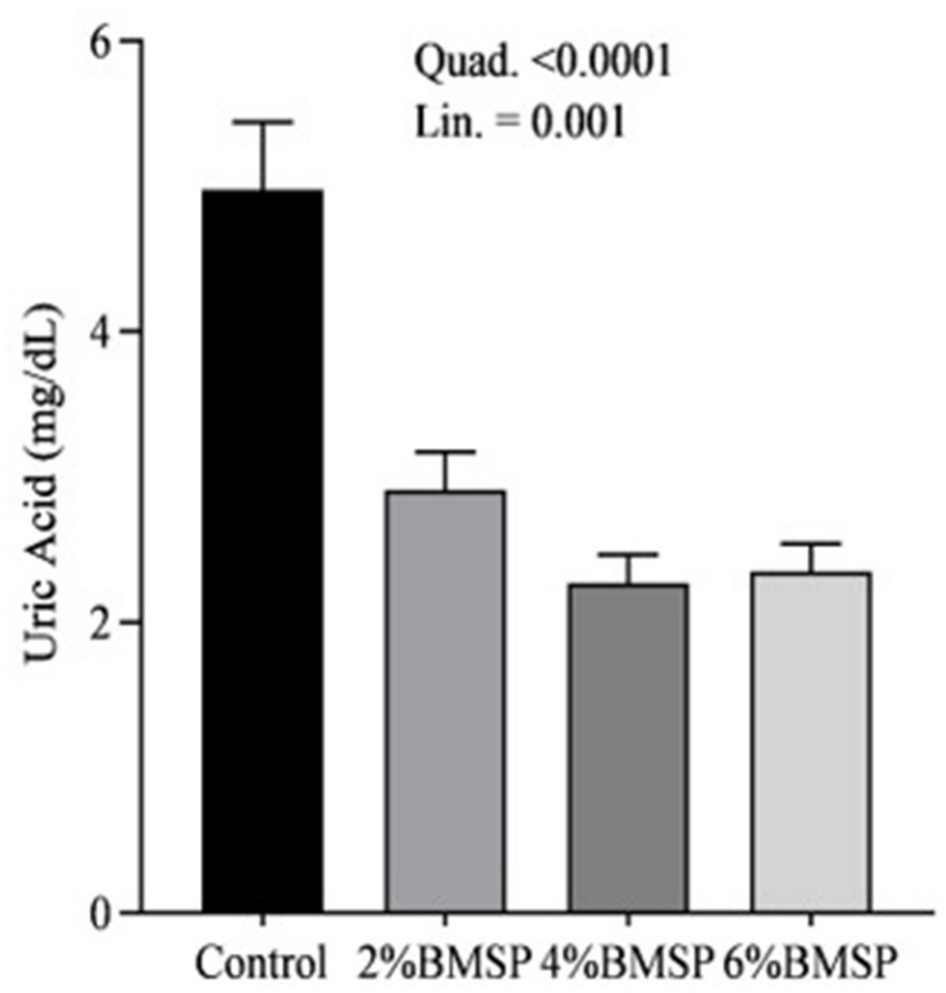
Effects of dietary brown mushroom stem powder (BMSP) supplementation on kidney function markers, including uric acid in layer chicks. Lin, linear response; Quad, quadratic response; 2% BMSP, 4% BMSP, 6% BMSP = diets containing 2%, 4%, or 6% BMSP, respectively.

**Figure 5:**
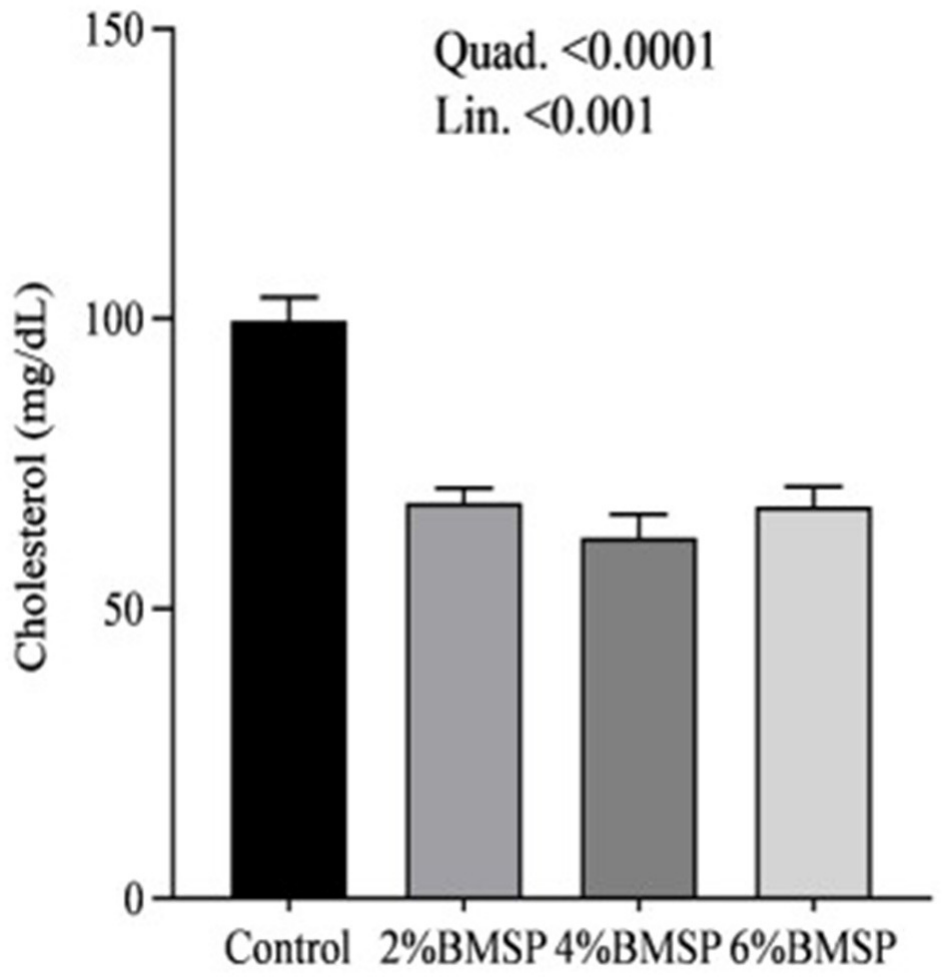
Effects of dietary brown mushroom stem powder (BMSP) supplementation on serum cholesterol concentrations in layer chicks. Lin, linear response; Quad, quadratic response; 2% BMSP, 4% BMSP, 6% BMSP, diets containing 2%, 4%, or 6% BMSP, respectively.

**Figure 6:**
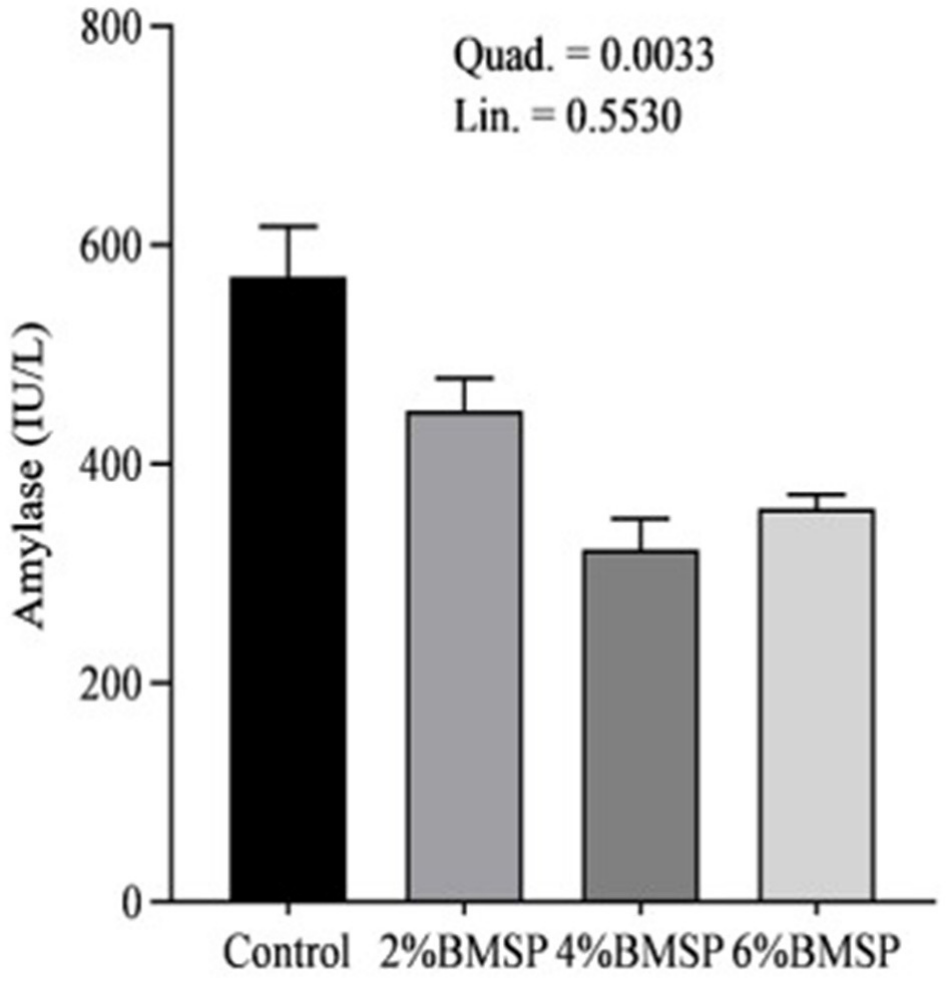
Effects of dietary brown mushroom stem powder (BMSP) supplementation on serum amylase activity in layer chicks. Lin, linear response; Quad, quadratic response; 2% BMSP, 4% BMSP, 6% BMSP, diets containing 2%, 4%, or 6% BMSP, respectively.

**Figure 7:**
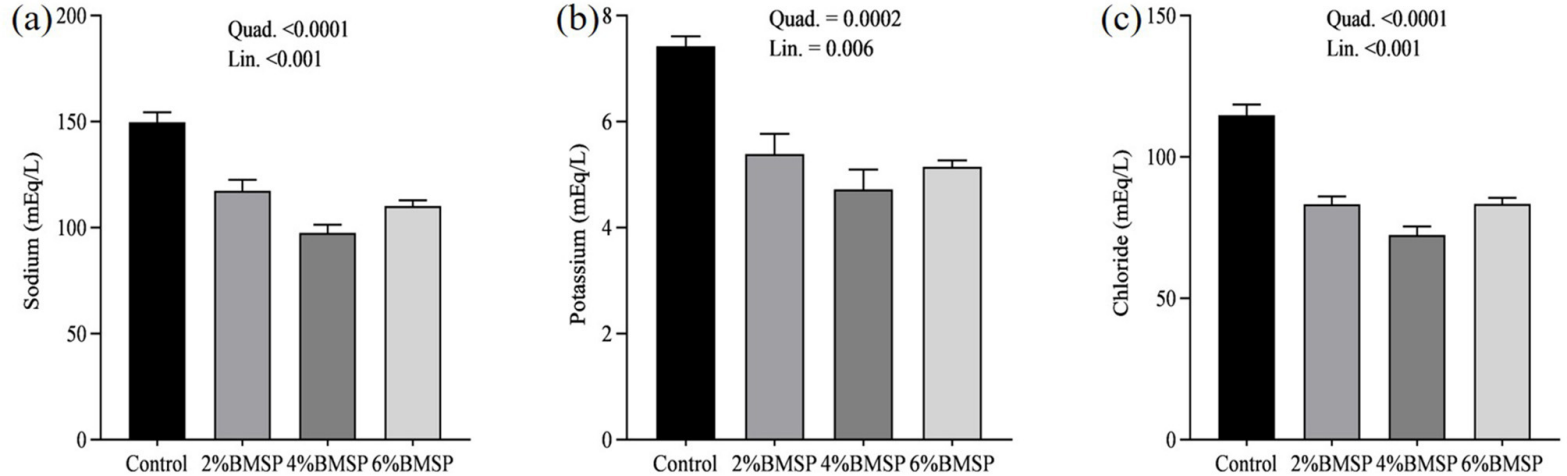
Effects of dietary brown mushroom stem powder (BMSP) supplementation on serum electrolyte levels, including sodium **(a)**, potassium **(b)**, and chloride **(c)**, in layer chicks. Lin, linear response; Quad, quadratic response; 2% BMSP, 4% BMSP, 6% BMSP, diets containing 2%, 4%, or 6% BMSP, respectively.

**Figure 8:**
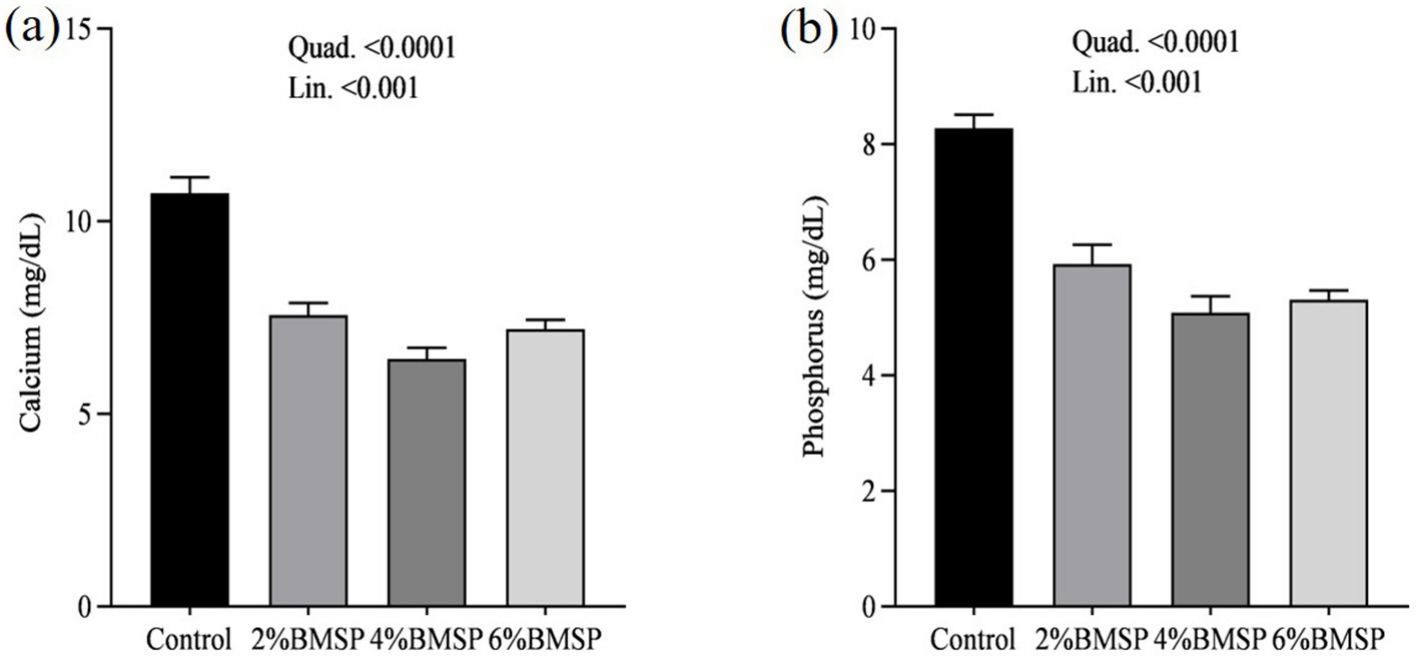
Effects of dietary brown mushroom stem powder (BMSP) supplementation on serum mineral concentrations, including calcium **(a)** and phosphorus **(b)**, in layer chicks. Lin, linear response; Quad, quadratic response; 2% BMSP, 4% BMSP, 6% BMSP, diets containing 2%, 4%, or 6% BMSP, respectively.

**Figure 9:**
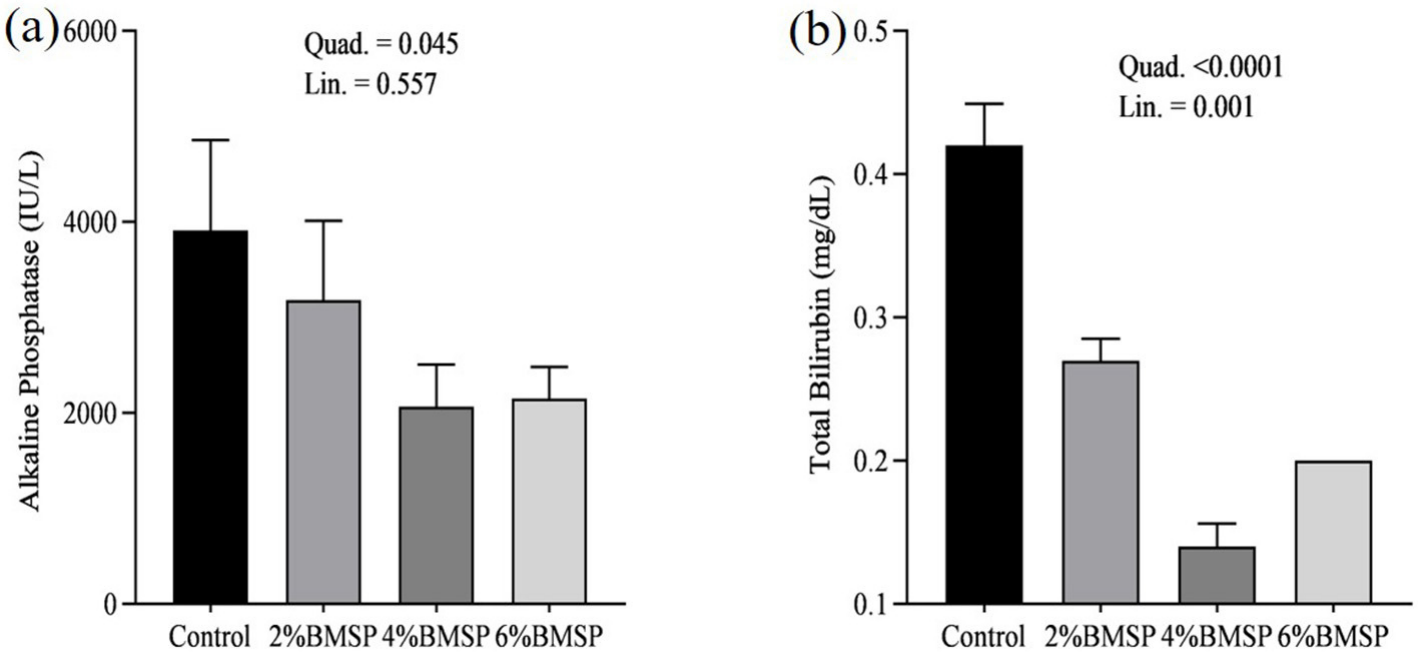
Effects of dietary brown mushroom stem powder (BMSP) supplementation on serum alkaline phosphatase **(a)** and total bilirubin **(b)** levels in layer chicks. Lin, linear response; 2% BMSP, 4% BMSP, 6% BMSP, diets containing 2%, 4%, or 6% BMSP, respectively.

The inclusion of BMS in the diet resulted in significant linear and quadratic decreases (*P* < 0.01) in serum proteins, including total protein and globulin (Figure 2), liver enzymes including aspartate aminotransferase and gamma-glutamyl transferase (Figure 3), kidney function markers including uric acid (Figure 4), lipid profile indicators such as cholesterol (Figure 5), and digestive enzymes (amylase, Figure 6). Similarly, serum electrolyte levels, including sodium, potassium, and chloride (Figure 7), as well as mineral markers such as calcium and phosphorus (Figure 8), also showed significant linear and quadratic responses to increasing BMS levels. Among these variables, only alkaline phosphatase and bilirubin (Figure 9) showed a significant linear trend without a corresponding quadratic response, while the remaining parameters demonstrated both trends, indicating a dose-dependent effect of BMS supplementation on the birds' biochemical profile.

### 3.4 Gas emission

**Figure 10:**
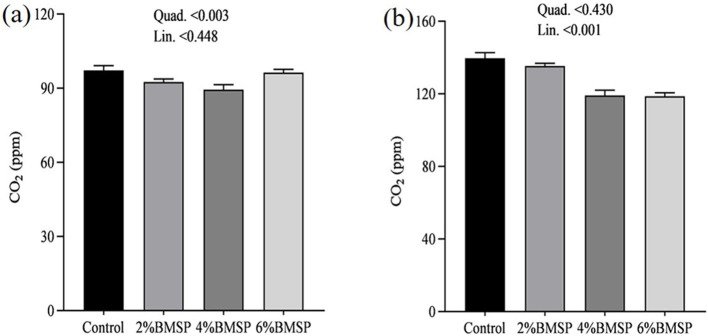
Effects of dietary brown mushroom stem powder (BMSP) supplementation on carbon dioxide (CO_2_) emissions in layer chicks measured on day 31 **(a)** and day 32 **(b)**. Lin, linear response; Quad, quadratic response; 2% BMSP, 4% BMSP, 6% BMSP, diets containing 2%, 4%, or 6% BMSP, respectively.

**Figure 11:**
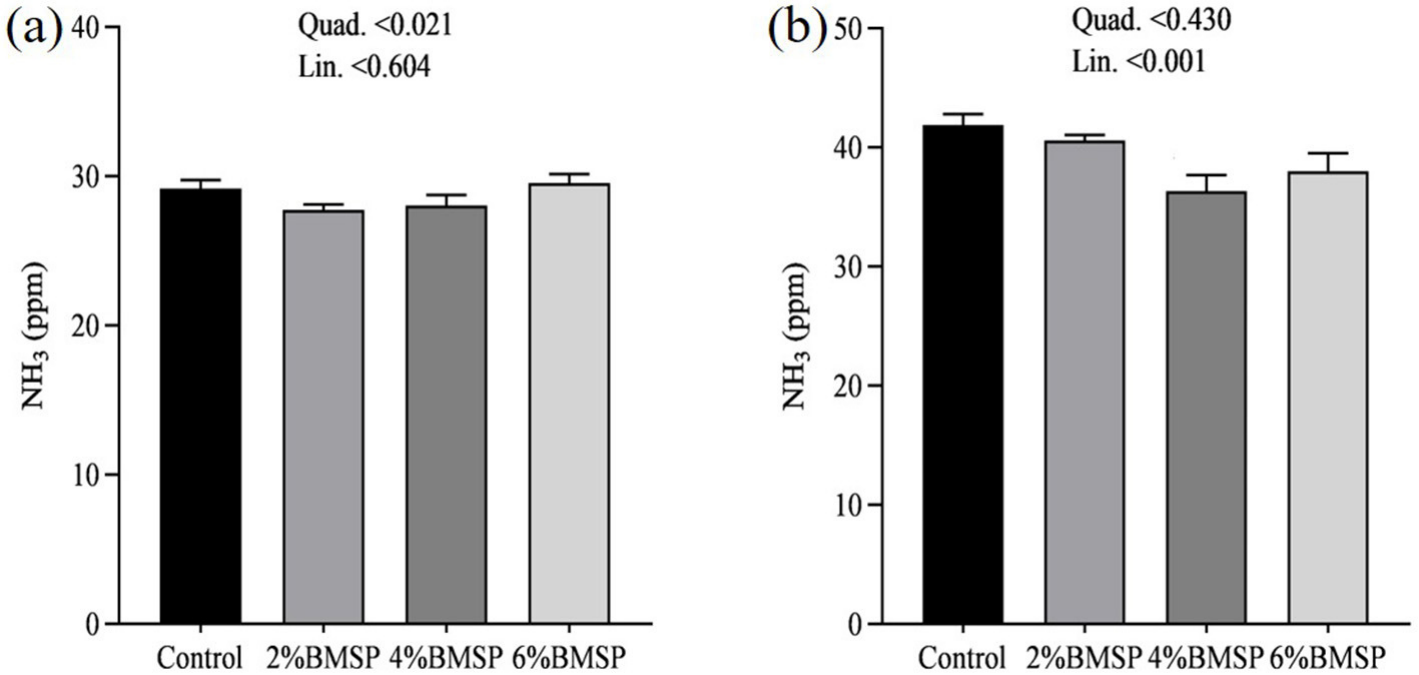
Effects of dietary brown mushroom stem powder (BMSP) supplementation on ammonia (NH_3_) emissions in layer chicks measured on day 31 **(a)** and day 32 **(b)**. Lin, linear response; Quad, quadratic response; 2% BMSP, 4% BMSP, 6% BMSP, diets containing 2%, 4%, or 6% BMSP, respectively.

**Figure 12:**
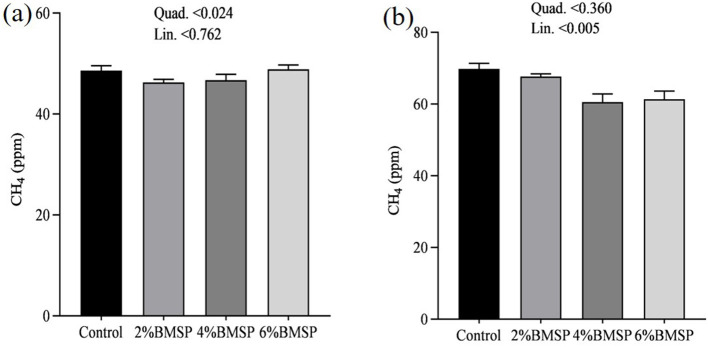
Effects of dietary brown mushroom stem powder (BMSP) supplementation on methane (CH_4_) emissions in layer chicks measured on day 31 **(a)** and day 32 **(b)**. Lin, linear response; Quad, quadratic response; 2% BMSP, 4% BMSP, 6% BMSP, diets containing 2%, 4%, or 6% BMSP, respectively.

On day 31, gas emissions showed quadratic changes, with CO_2_, NH_3_, and CH_4_ concentrations exhibiting non-linear patterns as BMS inclusion levels increased (Figures10a, 11a, 12a). Specifically, emissions decreased at the 2 and 4% BMS levels but plateaued or slightly increased at the 6% level. This suggests that early in the experimental period, the impact of BMS on gas emissions may be more variable. However, by day 32, the relationship between BMS inclusion and gas emissions shifted to linear trends, with CO_2_, NH_3_, and CH_4_ concentrations decreasing consistently as BMS levels increased (Figures10b, 11b, 12b). This indicates that over time, BMS supplementation led to a stable and predictable reduction in gas emissions. Overall, the results indicate that the inclusion of BMS in chick diets produces both quadratic and linear effects on gas emissions, depending on the duration of supplementation. The quadratic effects observed on day 31 highlight a more dynamic response early in the experiment, while the linear trends on day 32 suggest a consistent reduction in emissions over time. While the measurements were limited to a short 2-day period at the end of the trial, the results provide preliminary evidence that BMS inclusion may offer short-term environmental benefits. Further long-term monitoring is needed to confirm the stability and predictability of these effects over the full production cycle.

## 4 Discussion

The poultry industry is continuously seeking innovative and sustainable feed alternatives to reduce its dependence on conventional ingredients such as soybean meal, which is associated with environmental and economic concerns. BMS, a byproduct of button mushroom cultivation, holds considerable promise as a sustainable, eco-friendly, and cost-effective alternative. Rich in bioactive compounds, fiber, and essential nutrients (14, 15), BMS can contribute not only to feed sustainability but also to agricultural waste valorization, aligning with circular economy principles.

In recent years, mushroom-derived products have gained attention for their beneficial effects in poultry nutrition. Previous studies have shown the positive impacts of button mushroom supplementation on poultry growth, immunity, gut health, and antioxidant status (3, 16–18). Moreover, Altop et al. (19) found that stalk meal derived from *A. bisporus* could be safely included in broiler diets without detrimental effects on growth performance or carcass traits, supporting its potential as a feed component.

The results of this study indicate that replacing soybean meal up to 6% brown mushroom stem (BMS) powder in layer chick diets did not significantly affect growth performance. This lack of adverse impact may be attributed to the adequate crude protein and amino acids, and energy contribution of BMS, along with its digestible fiber and bioactive compounds that can enhance gut integrity and metabolic function. These properties collectively help maintain feed efficiency and overall performance, even when conventional protein sources are partially replaced.

The absence of adverse effects on performance could be attributed to BMS's nutritional and functional profile. Mushroom stems retain considerable quantities of polysaccharides, antioxidants, and other bioactive substances (14, 20), which may help maintain gut health and metabolic function even when partially replacing a high-protein ingredient like soybean meal. Furthermore, the stability of performance outcomes is in agreement with prior work demonstrating no adverse effects of mushroom powder supplementation on broiler or layer productivity when appropriately dosed (21, 22).

Collectively, these findings support the feasibility of using brown BMS as a sustainable feed ingredient in poultry diets. It offers a dual advantage: mitigating reliance on conventional protein sources and promoting the valorization of agricultural residues, thereby contributing to more resilient and environmentally friendly poultry production systems.

The components such as beta-glucans and mannans may contribute positively to gut health and nutrient utilization, potentially compensating for the slightly lower digestible nutrient content than conventional protein sources like soybean meal (4, 23). Furthermore, the non-significant changes in feed conversion ratio across all treatment groups suggest that the birds maintained efficient nutrient utilization regardless of BMS level. This could be partly due to the presence of some compounds in mushrooms, such as beta-glucans and mannans, which are known to modulate gut microbiota composition, improve intestinal integrity, and enhance nutrient absorption (24–26). Although growth performance metrics remained stable, such biofunctional effects may still provide long-term benefits to bird health, immunity, and productivity (27–29). Importantly, the use of BMS as a partial replacement for soybean meal also presents environmental and economic advantages. Mushroom stems are an agro-industrial byproduct, and their utilization in poultry diets aligns with circular economy principles by reducing feed costs and minimizing waste. The lack of adverse effects on growth performance further supports the feasibility of this sustainable alternative in layer chick diets. In summary, the results indicate that BMS can be used as a functional and eco-friendly feed ingredient in poultry nutrition without compromising growth performance, laying the foundation for future studies on its long-term health benefits with higher percentage (upto 30%) supplementation.

Regarding internal organ development, the present study demonstrates that dietary inclusion of BMS waste at levels up to 6% did not significantly alter the relative weights of key internal organs, including the liver, spleen, and gizzard, compared to the control group. These results suggest that BMS is a safe and physiologically well-tolerated feed component that does not adversely affect the growth or function of vital visceral organs in poultry. The absence of significant changes in liver and spleen weights is particularly noteworthy, as these organs serve as sensitive biomarkers for metabolic disturbances, immune system activation, and potential toxicological effects (5, 6). The consistency in their relative weights across all treatment groups suggests that BMS did not induce systemic stress or organ hypertrophy, reinforcing its non-toxic and biocompatible properties. Furthermore, the lack of variation in gizzard weight indicates that BMS supplementation did not alter the physical characteristics of the feed to a degree that would affect digestive organ adaptation. Interestingly, heart weight exhibited a significant linear increase with increasing levels of BMS in the diet. Although these changes remained within the physiological norm (24–26). These findings warrant further investigation, as the observed increase in heart weight may reflect a cardiovascular response to the functional bioactive compounds present in brown mushroom stem, particularly polyphenols and ergothioneine, which have been linked to improved cardiac efficiency and vascular health. However, to better understand the underlying mechanisms, future studies incorporating detailed histological analyses and assessments of relevant cardiac biomarkers are essential. Alternatively, this effect may represent an adaptive enhancement in cardiac output or circulatory function in response to subtle metabolic shifts promoted by BMS supplementation (30). Importantly, the observed increase in heart weight occurred independently of any adverse effects on growth performance or clinical health indicators, suggesting a physiological rather than pathological adaptation. Nevertheless, further investigations including histopathological and gene expression analyses are warranted to elucidate the underlying mechanisms and determine whether the observed changes reflect beneficial structural or functional modulation. Therefore, the inclusion of BMS in poultry diets appears safe and well-tolerated in terms of organ development. While the increase in heart weight merits further study, it may indicate a favorable cardiovascular response to the antioxidant-rich profile of mushroom stem byproducts.

The current findings demonstrate that dietary inclusion of BMS significantly affected several blood biochemical parameters in a dose-dependent manner, showing both linear and quadratic responses. Importantly, all observed changes remained within the normal physiological range (4, 16, 17). The observed decrease in serum total protein and globulin with increasing levels of brown mushroom stem may indicate improved protein utilization efficiency or a downregulation of immune-related protein synthesis (25, 26). This response could be attributed to the anti-inflammatory and immunomodulatory properties of bioactive compounds present in mushroom stems, such as beta-glucans and phenolic compounds, which may reduce systemic inflammation and lower antigenic stimulation. As a result, the demand for circulating globulins involved in immune defense could decline, reflecting a more efficient physiological state under dietary supplementation. Mushrooms are known to contain bioactive compounds such as polysaccharides, phenolics, and terpenoids, which have been reported to possess immunomodulatory properties (25, 26). These compounds may help reduce systemic inflammation, thereby decreasing the need for circulating globulins involved in immune defense. Liver enzymes, particularly aspartate aminotransferase and gamma-glutamyl transferase, also exhibited a decreasing trend, suggesting potential effects of mushroom-derived antioxidants (31–34). Brown mushroom stems are rich in phenolic compounds and ergothioneine, a unique antioxidant that is known to accumulate in animal tissues and protect hepatic cells from oxidative stress (35, 36). The reduction in these enzymes may indicate decreased hepatic cell turnover or improved liver function due to reduced lipid peroxidation (37). The significant decline in uric acid and cholesterol levels suggests an improvement in renal excretion efficiency and lipid metabolism, which is consistent with the reported hypolipidemic effects of mushroom-derived bioactive compounds (38, 39). Mushrooms are a known source of dietary fiber and beta-glucans, which have cholesterol-lowering properties by binding bile acids and promoting their excretion (40). Moreover, the hypouricemic effect might be attributed to the presence of phenolic compounds that inhibit xanthine oxidase activity, thereby reducing uric acid synthesis (41). Electrolyte balance was also influenced by mushroom stem supplementation, with reductions in sodium, potassium, and chloride concentrations. This may be due to enhanced water and mineral retention mechanisms or a shift in renal reabsorption and excretion dynamics influenced by dietary fibers and antioxidants (42). The alteration in mineral markers such as calcium and phosphorus could be linked to improved absorption or altered bone metabolism due to the bioavailability of certain mushroom-derived micronutrients (19). The overall pattern of changes in biochemical markers suggests that brown mushroom stem exerts multiple physiological effects, likely through its antioxidant, anti-inflammatory, and hypolipidemic properties (36). The dose-dependent nature of these effects also highlights the potential of brown mushroom stems as a functional feed additive capable of improving metabolic health and nutrient utilization in poultry.

The inclusion of BMS in poultry diets demonstrated significant effects on gas emissions, with both quadratic and linear responses observed over the course of the study. Specifically, on day 31, the relationship between BMS inclusion levels and gas emissions was more dynamic, showing non-linear trends for CO_2_, NH_3_, and CH_4_. Emissions decreased at the 2 and 4% BMS levels but plateaued or slightly increased at the 6% inclusion level. By day 32, however, the gas emission trends shifted to linear, with a consistent reduction in CO_2_, NH_3_, and CH_4_ concentrations as BMS levels increased. Although gas emissions were measured only on days 31 and 32 due to equipment availability, the controlled conditions under which measurements were taken provide meaningful preliminary insights. Nonetheless, future studies with continuous monitoring are necessary to confirm these trends over the full production cycle. Several potential mechanisms can explain the observed reductions in gas emissions. One key factor is the improved nitrogen utilization resulting from BMS inclusion. BMS may enhance protein digestibility or alter nitrogen metabolism, leading to reduced nitrogen excretion and consequently lower ammonia and ammonium emissions. The bioactive compounds in BMS, such as polyphenols and ergothioneine, may also exert antioxidant and anti-inflammatory effects within the gastrointestinal tract, promoting a healthier gut environment and reducing gut inflammation, which can subsequently lower the production of nitrogenous waste (35, 43, 44).

Another important factor is the prebiotic potential of BMS, which is rich in dietary fibers such as β-glucans and chitin. These fibers can selectively promote beneficial gut microbiota while suppressing proteolytic and ureolytic bacteria responsible for NH_3_ production. This microbial shift may help explain the reduced gas emissions observed in the study. Furthermore, BMS may enhance nutrient digestibility in the upper gastrointestinal tract, thereby reducing protein fermentation in the hindgut and further decreasing the production of gaseous by-products like CO_2_ and NH_3_ (24–26).

The reduction in gas emissions, particularly NH_3_, has important implications for both the environmental sustainability and animal welfare of poultry production (45). NH_3_ is a major pollutant in poultry barns, contributing to air and soil acidification, and it has been linked to respiratory problems and stress in poultry. By decreasing NH_3_ emissions, BMS not only helps mitigate the environmental footprint of poultry farming but also improves air quality within poultry houses, leading to better respiratory health, reduced stress, and potentially improved performance in birds (46, 47). These benefits are particularly important for maintaining optimal growth conditions and reducing mortality in commercial poultry production. In addition to the environmental and welfare advantages, the inclusion of BMS in poultry diets offers economic benefits. Lower NH_3_ concentrations can reduce the need for expensive ventilation systems, thus lowering operational costs for poultry farms (47). Furthermore, BMS, as an agricultural by-product, can provide a sustainable and cost-effective alternative to conventional feed ingredients like soybean meal, which is subject to fluctuating market prices. This makes BMS an attractive option for integrating circular economy principles into poultry production, reducing waste while enhancing feed efficiency (48). So, including BMS in poultry diets shows promising potential to reduce gas emissions, improve environmental sustainability, and support animal welfare. These findings suggest that BMS may be a valuable ingredient in developing more eco-friendly and efficient poultry production systems. Future studies should focus on further elucidating the microbial and molecular mechanisms behind these effects, and explore the long-term implications of BMS inclusion in commercial poultry operations.

## 5 Conclusion

Brown mushroom stem powder shows promising potential as a sustainable and eco-friendly alternative to soybean meal in poultry diets. Inclusion levels of up to 6% supported comparable growth performance and internal organ development, while also contributing to reduced gas emissions and favorable modulation of blood biochemical parameters,. These findings suggest that BMS could serve as a functional feed ingredient aligned with the objectives of sustainable animal agriculture. However, as this study was limited to the grower phase, it did not assess production-phase parameters such as egg traits, long-term health effects, or feed cost-effectiveness in laying hens. Therefore, this work should be regarded as an initial, exploratory investigation. Further long-term studies under commercial conditions are essential to evaluate the practical relevance of BMS supplementation on layer performance, egg quality, economic viability, and overall sustainability.

## Data Availability

The raw data supporting the conclusions of this article will be made available by the authors, without undue reservation.
